# Severe Respiratory Distress in a Child with Pulmonary Idiopathic Hemosiderosis Initially Presenting with Iron-Deficiency Anemia

**DOI:** 10.1155/2015/876904

**Published:** 2015-11-08

**Authors:** A. Potalivo, L. Finessi, F. Facondini, A. Lupo, C. Andreoni, G. Giuliani, C. Cavicchi

**Affiliations:** Department of Emergency, Anaesthesia and Intensive Care Section, Infermi Hospital, Viale Luigi Settembrini 2, 47923 Rimini, Italy

## Abstract

Idiopathic pulmonary hemosiderosis (IPH) is a rare cause of alveolar hemorrhage in children but should be considered in children with anemia of unknown origin who develop respiratory complications. It is commonly characterized by the triad of recurrent hemoptysis, diffuse parenchymal infiltrates, and iron-deficiency anemia. Pathogenesis is unclear and diagnosis may be difficult along with a variable clinical course. A 6-year-old boy was admitted to the hospital with a severe iron-deficiency anemia, but he later developed severe acute respiratory failure and hemoptysis requiring intubation and mechanical ventilation. The suspicion of IPH led to the use of immunosuppressive therapy with high dose of corticosteroids with rapid improvement in clinical condition and discharge from hospital.

## 1. Background

Idiopathic pulmonary hemosiderosis (IPH) is a rare cause of alveolar hemorrhage in children [1, 2]. It is commonly characterized by the triad of recurrent hemoptysis, diffuse parenchymal infiltrates, and iron-deficiency anemia [3–5]. Pathogenesis is unclear and diagnosis may be difficult due to a variable clinical course [5, 6]. About 500 cases of this disease have been described in medical literature [5, 7]. IPH is usually a diagnosis of exclusion as not one identifying test has been described [6]. Currently used intensive care therapies include high dose steroid and immunosuppressive treatment along with conventional and high-frequency oscillatory ventilation. In children who cannot maintain adequate oxygenation with conventional therapies extracorporeal life support has been described [6, 8, 9]. The aim of this paper is to present the diagnostic challenge and intensive care unit management of a 6-year-old boy with a severe respiratory failure due to IPH initially presenting as an iron-deficiency anemia.

## 2. Case Presentation

**Figure 1:**
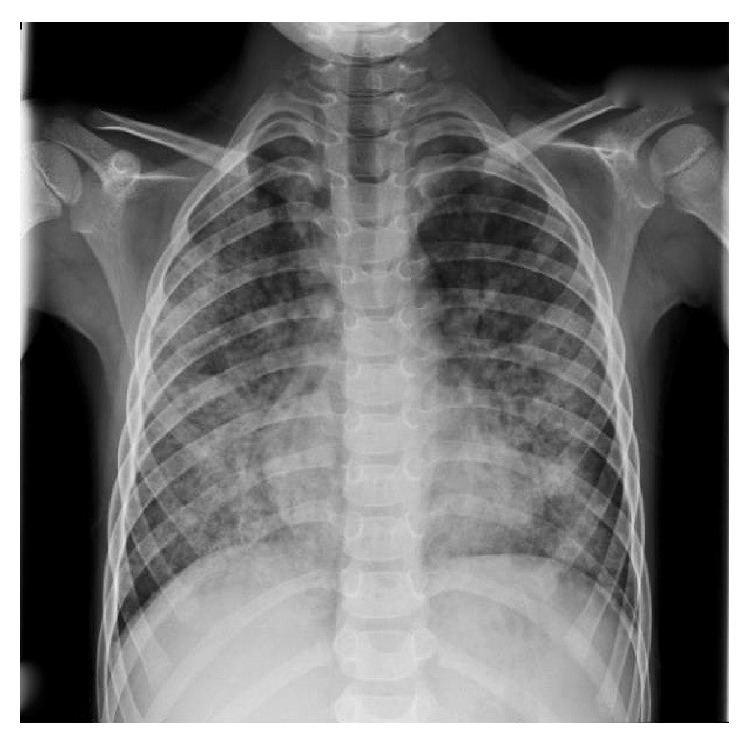
Posteroanterior chest radiography showing diffuse bilateral pulmonary infiltrations.

A 6-year-old boy of 20 kg weight was admitted to the hospital, with a recent history of progressive paleness and general fatigue. The patient was alert, with profound dyspnea, and unable to maintain oxygen saturation in room air (SaO_2_ < 80%); his cardiac frequency was 130 bpm and BP was 90/40 mmHg. Physical examination was positive for skin and mucous membrane pallor. The chest radiograph was positive for multiple alveolar-type opacities with a background of interstitial reticular pattern (Figure 1). History was positive for previous tonsillectomy and familiar cases of celiac disease. One month before the child was again hospitalized for severe anemia requiring blood transfusions, laboratory investigations showed severe anemia with hemoglobin (Hb) 4.6 g/dL, microcytosis, and hypocromia with level of serum iron and transferritin decreased. His vital signs were normal; bleeding from gastrointestinal tract was excluded and bone marrow biopsy showed nonspecific findings of dyserythropoiesis. Serologic studies were negative. He was discharged from hospital but subsequent follow-up showed persistent anemia despite iron therapy and several blood transfusion with packet red blood cell units.

When he was readmitted to emergency department he was febrile (37.9°C) with severe respiratory distress; laboratory confirmed persistent anemia and elevated inflammatory indices (white blood cells 18.830 × 10^3^/*μ*L and C-reactive protein of 21.9 mg/L).

**Figure 2:**
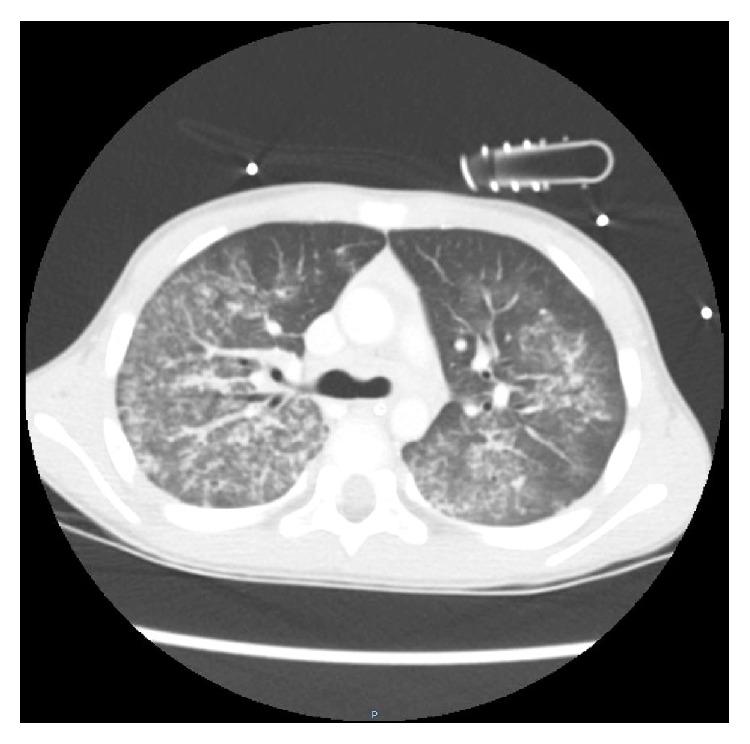
Chest computed tomography shows areas of ground-glass attenuation and a reticular micronodular appearance in both lung fields.

**Figure 3:**
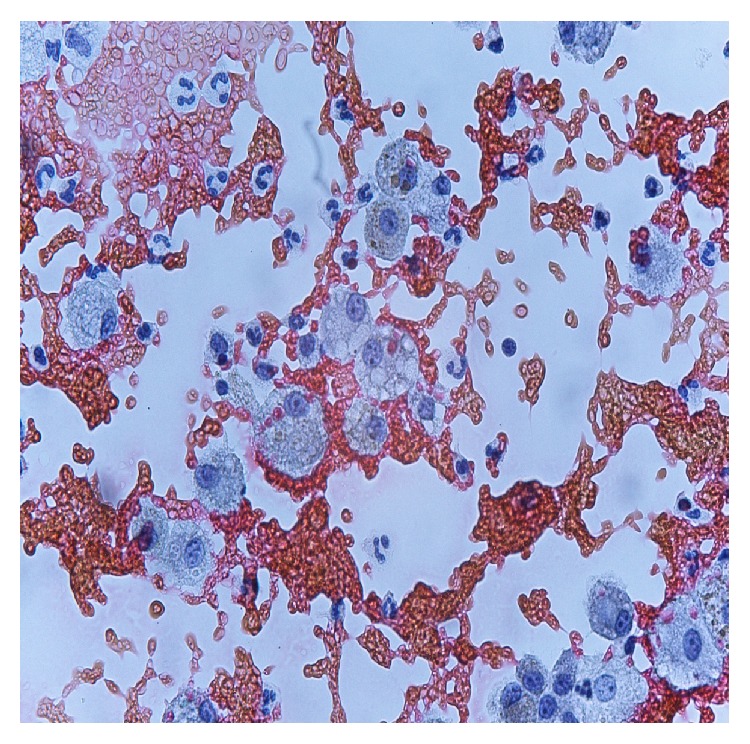
BAL specimen showing hemosiderin-laden macrophages. Staining for iron (Perls' Prussian blue). Magnification ×400.

He was transferred to the ICU after starting broad spectrum antibiotics with suspicion of severe sepsis or transfusion related acute lung injury. Few hours later severe hemoptysis occurred and a diagnosis of IPH was supposed. The patient was treated with bolus infusion of methilprednisolone 10 mg/kg and subsequentially 20 mg prednisone four times daily, but worsening respiratory failure required endotracheal intubation and mechanical ventilation. He was sedated with infusion of Propofol 6 mg/kg/h and Remifentanyl 0.05 *μ*g/kg/min and he was ventilated with protective strategy using low tidal volume values and positive end-expiratory pressure of 5 cmH_2_O due to severe respiratory distress (PaO_2_/FiO_2_ ratio of 90). Rapid clinical improvement was noted and the following day a CT scan showed diffuse alveolar consolidation compatible with a recent bleeding (Figure 2). A bronchoscopy was performed and microscopic examination of the bronchoalveolar lavage fluid revealed the presence of many hemosiderin-laden macrophages (Figure 3) with no evidence of infection. Laboratory work-up including antinuclear antibodies (ANA), anti-neutrophil cytoplasmic antibodies (ANCA), extractable nuclear antigens (ENA) antibodies, rheumatoid factor, antigliadin, tissue transglutaminase antibody IgA class, antiglomerular basement membrane (antiGBM), and specific cow's milk IgE and complement was negative. Cyclic citrullinated peptide antibodies (anti-CCP) were also negative. Improvement in gas exchanges led to extubation four days later. Noninvasive ventilation support was started due to persistence of mild respiratory distress. The child was transferred to medical ward after eight days of ICU stay and then discharged from hospital after other nine days. Prolonged follow-up showed good clinical recovery with methilprednisolone pulses of 30 mg/kg for three days and repeated monthly. During progressive tapering off of corticosteroids the child suffered of another self-limiting episode of hemoptysis without sequelae. For this reason current daily maintaining dose of oral prednisone is 1 mg/kg/day and methilprednisolone pulses were resumed. Despite negative serologic findings, the child followed a gluten-free diet without any apparently long term benefit. There was not any adverse effect of corticosteroid treatment. Outcome is satisfactory, but patient's quality of life got worse due to exertional dyspnea.

## 3. Discussion

Idiopathic pulmonary hemosiderosis is a rare and life threatening type of diffuse alveolar hemorrhage (DAH) that preferentially affects children and young adults [1, 4, 5]. Pathophysiology of the disease is complex and elusive and its etiology remains unknown. Allergic, environmental, genetic, and/or autoimmune hypotheses have been proposed to explain the structural lesions of alveolar-endothelial membrane seen in IPH [1]. While there is no definitive etiology of IPH, an underlying immune process is likely, given its typical responsiveness to immunosuppressive therapy [10, 11].

Estimated pediatric IPH incidence is 0.24 and 1.23 cases per million, with a mortality rate as high as 50% in previous reports [11, 12]. It commonly occurs in the ages of 1–7 years [13], but 20% of cases are adult-onset patients [4]. It is classically characterized by a triad of hemoptysis, iron-deficiency anemia, and pulmonary infiltrates [4, 5, 14]. However, any of these features may be the first presenting manifestation, so the diagnosis of IPH can be difficult [11]. In previous reports the classic triad was found only in 15% to 26% of patients diagnosed with IPH [15, 16]. Anemia and dyspnea are the most frequent clinical features and hemoptysis occurs in about 50% of patients [3], but its incidence could be underestimated in young children, who frequently swallow their sputum [4].

In our case iron-deficiency anemia preceded other symptoms and signs, so the diagnosis of IPH was delayed. Pulmonary hemorrhages and hemoptysis are rare in children and the absence of respiratory symptoms led us to look for other causes of iron-deficiency anemia like cystic fibrosis, congenital heart diseases, malignancies, and gastrointestinal or laryngeal pathologies [5, 17]. It should be stressed to consider the diagnosis of a rare pathology like IPH when there is no response to iron therapy and blood transfusions and no clear site of bleeding [4, 18]. Dyspnea and hemoptysis along with severe respiratory distress that occurred with second clinical presentation drew our attention to a possible DAH.

DAH syndromes are heterogeneous pathologies taking place as a result of injury to the small lung vessels (capillaries, but also arterioles and venules) and they can occur with or without pulmonary capillaritis [19]. IPH is a disorder where there is no cardiovascular cause and no evidence of capillaritis. Renal and systemic manifestations are absent. There are reports of patients, initially diagnosed with IPH, who are later found to have Goodpasture's syndrome, systemic lupus erythematosus, or microscopic polyangiitis. IPH should be considered a diagnosis of exclusion [19, 20] as in our case where all serologic findings were negative.

The lung biopsy still remains the gold standard for definite diagnosis, but IPH can also be confirmed by bronchoscopy with bronchoalveolar lavage showing hemosiderin-laden macrophages [1–5].

We decided not to perform lung biopsy, due to severe respiratory distress and given the fact that the bronchoalveolar lavage was highly suggestive.

Interestingly there was a positive familiar history of celiac disease in our patient. IPH has frequently been associated with celiac disease. This association is well-known as the Lane-Hamilton syndrome and there are reports of positive respiratory outcome with a gluten-free diet [21, 22]. Some authors suggest to systematically perform gastrointestinal endoscopies and biopsies in IPH patients, even in the absence of gastrointestinal symptoms, when the severity of anemia is disproportionate to radiologic findings [21, 23]. We screened for specific antibodies of celiac disease (antigliadin and anti-tranglutaminase antibodies), but they were negative so we did not request HLA types. However the child did not apparently benefit of a gluten-free diet.

It is essential to make a prompt diagnosis in order to avoid complications of recurrent alveolar hemorrhages like interstitial fibrosis [4, 10, 11]. High resolution CT scan is useful for early detection of pulmonary fibrosis [10, 24], and it helped us to exclude this complication.

Pulmonary function testing techniques are well established in children and adolescents. However children aged 2–6 years represent a real challenge in pulmonary function assessment due to lack in cooperation. Lung diffusing capacity of carbon monoxide (DLCO) is often markedly reduced and may be abnormal before any radiologic abnormalities [2].

There are no evidence-based recommendations regarding treatment for acute onset DAH and in particular for IPH [1, 3–5, 10]. Corticosteroids have long been used for treatment of IPH [3, 11]. Their use is associated with a decrease of the frequency of hemorrhages, although it is not known if they have any effect on the course of the disease and progression to pulmonary fibrosis [4]. Immunosoppressive therapy has also been used, especially in cases of steroid-dependence or steroid-resistance diseases [1, 11, 14]. Among immunosuppressant agents, azathioprine in combination with corticosteroids might be the best therapeutic regimen, especially in preventing IPH exacerbations [1, 4]. In our cases corticosteroids therapy was effective and clinical outcome was satisfactory. Only few cases of single-lung transplantation have been reported as a therapy of end stage IPH, with failure of this therapeutic option due to reoccurrence of the disease [25, 26]. Long term follow-up should take into account the numbers and severity of hemorrhagic episodes and the progression of interstitial disease (as expressed by the decline of DLCO) [1]. Fortunately the prognosis of IPH seems to improve over time. Two decades ago the mean survival was 3 years, but recent data indicate a 5-year survival of 86% of cases possibly due to the long term use of immunosuppressant therapy [4, 5, 11].

Respiratory distress in our patient was particularly severe leading to intubation and mechanical ventilation. Little is known about need for ventilatory support in pediatric patients with severe IPH. Rabe and coworkers report a series of 37 adult patients with DAH admitted to ICU for severe respiratory distress. Eighty-six percent of them (32 patients) were mechanically ventilated [27]. Sun and coworkers described a 11-year-old case of pediatric IPH leading to ARDS and ventilatory support [9]. In their case conventional ventilatory support failed to maintain adequate respiratory gas exchanges so extracorporeal membrane oxygenation (ECMO) was started. Another case of extracorporeal life support in a 5-week-old infant with IPH has been described with good clinical outcome [6]. Our case did not require ECMO given the fact that respiratory gas exchanges rapidly ameliorated after starting corticosteroid therapy.

Although mechanical ventilation could be life-saving in these situations, it is essential to limit the possibility of ventilation induced lung injury (VILI) [28]. Artificial lung ventilation can further damage the alveolar-endothelial membrane, so it is recommended to limit tidal volume to 4–6 mL/kg and give positive end-expiratory alveolar pressure (PEEP) in order to limit cyclic collapse and opening of terminal airways during tidal ventilation [29]. Increasing the PEEP during mechanical ventilation may otherwise produce a tamponade effect to limit capillary bleeding from disrupted alveolar-capillary membranes [19].

Our case emphasizes the importance for the respiratory physician to consider IPH as a possible diagnosis when a child with idiopathic anaemia develops severe respiratory failure, in order to avoid the possible long term sequelae of untreated disease.

## Abbreviations

DAH: Diffuse alveolar hemorrhage
IPH: Idiopathic pulmonary hemosiderosis
Hb: Haemoglobin
ICU: Intensive care unit
ANA: Antinuclear antibodies
ANCA: Anti-neutrophil cytoplasmic antibodies
ENA: Extractable nuclear antigens
GBM: Glomerular basement membrane
ARDS: Acute respiratory distress syndrome
VILI: Ventilation induced lung injury
DLCO: Diffusing capacity for carbon monoxide
PEEP: Positive end-expiratory pressure.

## References

[1] Ioachimescu O. C., Sieber S., Kotch A. (2004). Idiopathic pulmonary haemosiderosis revisited. *European Respiratory Journal*.

[2] Clement A., Nathan N., Epaud R., Fauroux B., Corvol H. (2010). Interstitial lung diseases in children. *Orphanet Journal of Rare Diseases*.

[3] Taytard J., Nathan N., de Blic J. (2013). New insights into pediatric idiopathic pulmonary hemosiderosis: the French RespiRare cohort. *Orphanet Journal of Rare Diseases*.

[4] Bakalli I., Kota L., Sala D. (2014). Idiopathic pulmonary hemosiderosis—a diagnostic challenge. *Italian Journal of Pediatrics*.

[5] Kamienska E., Urasinski T., Gawlikowska-Sroka A., Glura B., Pogorzelski A. (2009). Idiopathic pulmonary hemosiderosis in a 9-year-old girl. *European Journal of Medical Research*.

[6] Gutierrez S., Shaw S., Huseni S. (2014). Extracorporeal life support for a 5-week-old infant with idiopathic pulmonary hemosiderosis. *European Journal of Pediatrics*.

[7] Sawielajc K., Krus J., Balcar-Boron A. (1994). Spontaneous pulmonary hemosiderosis in a four-year-old boy. *Wiad Lek*.

[8] Kolovos N. S., Schuerer D. J. E., Moler F. W. (2002). Extracorporal life support for pulmonary hemorrhage in children: a case series. *Critical Care Medicine*.

[9] Sun L.-C., Tseng Y.-R., Huang S.-C. (2006). Extracorporeal membrane oxygenation to rescue profound pulmonary hemorrhage due to idiopathic pulmonary hemosiderosis in a child. *Pediatric Pulmonology*.

[10] Milman N., Pedersen F. M. (1998). Idiopathic pulmonary haemosiderosis. Epidemiology, pathogenic aspects and diagnosis. *Respiratory Medicine*.

[11] Saeed M. M., Woo M. S., MacLaughlin E. F., Margetis M. F., Keens T. G. (1999). Prognosis in pediatric idiopathic pulmonary hemosiderosis. *Chest*.

[12] Soergel K. H., Sommers S. C. (1962). Idiopathic pulmonary hemosiderosis and related syndromes. *The American Journal of Medicine*.

[13] Heiner D. C., Chernick V., Kendig E. L. (1990). Pulmonary hemosiderosis. *Disorders of the Respiratory Tract in Children*.

[14] Willms H., Gutjahr K., Juergens U. R. (2007). Diagnostics and therapy of idiopathic pulmonary hemosiderosis. *Medizinische Klinik*.

[15] Bulucea C., Sorin D. (2008). Idiopathic pulmonary hemosiderosis in children: a Romanian experience. *Pediatrics*.

[16] Kabra S. K., Bhargava S., Lodha R., Satyavani A., Walia M. (2007). Idiopathic pulmonary hemosiderosis: clinical profile and follow up of 26 children. *Indian Pediatrics*.

[17] Godfrey S. (2004). Pulmonary hemorrhage/hemoptysis in children. *Pediatric Pulmonology*.

[18] Sankararaman S., Shah K., Maddox K., Velayuthan S., Scott L. K. (2012). Clinical case of the month. Idiopathic pulmonary hemosiderosis presenting as a rare cause of iron deficiency anemia in a toddler—a diagnostic challenge. *The Journal of the Louisiana State Medical Society*.

[19] Susarla S. C., Fan L. L. (2007). Diffuse alveolar hemorrhage syndromes in children. *Current Opinion in Pediatrics*.

[20] Serisier D. J., Wong R. C. W., Armstrong J. G. (2006). Alveolar haemorrhage in anti-glomerular basement membrane disease without detectable antibodies by conventional assays. *Thorax*.

[21] Mayes D. H., Guerrero M. L. (2008). A few good men: a marine with hemoptysis and diarrhea. *Chest*.

[22] Sethi G. R., Singhal K. K., Puri A. S., Mantan M. (2011). Benefit of gluten-free diet in idiopathic pulmonary hemosiderosis in association with celiac disease. *Pediatric Pulmonology*.

[23] Keskin O., Keskin M., Guler E. (2011). Unusual presentation: pulmonary hemosiderosis with celiac disease and retinitis pigmentosa in a child. *Pediatric Pulmonology*.

[24] Buschman D. L., Ballard R. (1993). Progressive massive fibrosis associated with idiopathic pulmonary hemosiderosis. *Chest*.

[25] Calabrese F., Giacometti C., Rea F. (2002). Recurrence of idiopathic pulmonary hemosiderosis in a young adult patient after bilateral single-lung transplantation. *Transplantation*.

[26] Wroblewski B. M., Stefanovic C. R., McDonough V. M., Kidik P. J. (1997). The challenges of idiopathic pulmonary hemosiderosis and lung transplantation. *Critical Care Nurse*.

[27] Rabe C., Appenrodt B., Hoff C. (2010). Severe respiratory failure due to diffuse alveolar hemorrhage: clinical characteristics and outcome of intensive care. *Journal of Critical Care*.

[28] Dreyfuss D., Saumon G. (1998). Ventilator-induced lung injury lessons from experimental studies. *American Journal of Respiratory and Critical Care Medicine*.

[29] Hess D. R. (2011). Approaches to conventional mechanical ventilation of the patient with acute respiratory distress syndrome. *Respiratory care*.

